# Induction of HIV mucosal immunity at distal sites after encapsulation of NOD1 and NOD2 ligands in biodegradable nanocarriers

**DOI:** 10.1186/1742-4690-9-S2-P194

**Published:** 2012-09-13

**Authors:** V Pavot, N Rochereau, V Lahaye, C Genin, E Perouzel, T Lioux, C Primard, T Delair, S Paul, B Verrier

**Affiliations:** 1 IBCP-CNRS, Lyon, France; 2 GIMAP, Saint Etienne, France; 3 Cayla Invivogen, Toulouse, France; 4 University of Lyon - IMP, Lyon, France

## Background

The use of TLR ligands as mucosal adjuvant for vaccine administration is already largely described; wherease the use of NOD-like receptors ligands is still investigated. As activation of intracytoplasmic NOD like-receptors is able to induce production of pro-inflammatory molecules, we have evaluated if their co-delivery into biodegradable nanocarriers carrying HIV-Gag antigens could amplify the mucosal immune responses in mice at the vaginal and intestinal (HIV replication site)

## Methods

We used Poly(Lactic Acid) (PLA) nanoparticles (NPs) (~200 nm) for co-delivery of p24 and NOD ligands. As NOD like-receptors are mainly expressed by antigen presenting cells, we first assessed the capacity of free or encapsulated ligands to induce monocyte derived dendritic cells (MoDCs) maturation. Then, as NOD like-receptors are principally expressed at the intestinal level we compared by oral immunization of BALB/c mice encapsulated ligands co-delivered with PLA-p24 NPs. To assess the adjuvant effect, p24-specific cellular and humoral responses were analyzed on splenocytes and in vaginal washes, faeces and sera.

## Results

The state of MoDCs maturation was characterized by the expression of CD80, CD83 and CD86. We showed that encapsulation of NOD1 or NOD2 ligand increases significantly their expression, compared to the effect of free ligands, probably due to a better uptake of encapsulated ligands.

By analyzing humoral immune responses, we observed that co-administration of p24 and NOD2 ligand by two different NPs was the most efficient formulation to induce anti-p24 IgG and IgA responses in faeces. By contrast co-formulation of p24 and NOD1 ligand in the same NP induced a better CD8 IFNγ response.

## Conclusion

Encapsulation of NOD ligands into PLA nanoparticles seems to favour their action on DCs maturation, their co-administration with PLA-p24 NPs inducing an adjuvant effect. Use of those ligands as mucosal adjuvant deserve further experiments and we are investigating the mechanisms involved and increasing delivery efficacy after co-encapsulations.

